# Online and offline effects of transcranial alternating current stimulation of the primary motor cortex

**DOI:** 10.1038/s41598-021-83449-w

**Published:** 2021-02-16

**Authors:** Ivan Pozdniakov, Alicia Nunez Vorobiova, Giulia Galli, Simone Rossi, Matteo Feurra

**Affiliations:** 1 Centre for Cognition and Decision Making, Institute for Cognitive Neuroscience, National Research University, Higher School of Economics, Myasnitskaya St. 20, 101000 Moscow, Russia; 2 Department of Psychology, Kingston University, Penrhyn Road, Kingston Upon Thames, KT1 2EE UK; 3 Department of Medicine, Surgery and Neuroscience, Siena Brain Investigation & Neuromodulation Lab (Si-BIN Lab.), Unit of Neurology and Clinical Neurophysiology and Section of Human Physiology, University of Siena, 53100 Siena, Italy; 4 National Research University, Higher School of Economics, 101000 Moscow, Russia

**Keywords:** Motor cortex, Neuronal physiology

## Abstract

Transcranial alternating current stimulation (tACS) is a non-invasive brain stimulation technique that allows interaction with endogenous cortical oscillatory rhythms by means of external sinusoidal potentials. The physiological mechanisms underlying tACS effects are still under debate. Whereas online (e.g., ongoing) tACS over the motor cortex induces robust state-, phase- and frequency-dependent effects on cortical excitability, the offline effects (i.e. after-effects) of tACS are less clear. Here, we explored online and offline effects of tACS in two single-blind, sham-controlled experiments. In both experiments we used neuronavigated transcranial magnetic stimulation (TMS) of the primary motor cortex (M1) as a probe to index changes of cortical excitability and delivered M1 tACS at 10 Hz (alpha), 20 Hz (beta) and sham (30 s of low-frequency transcranial random noise stimulation; tRNS). Corticospinal excitability was measured by single pulse TMS-induced motor evoked potentials (MEPs). tACS was delivered online in Experiment 1 and offline in Experiment 2. In Experiment 1, the increase of MEPs size was maximal with the 20 Hz stimulation, however in Experiment 2 neither the 10 Hz nor the 20 Hz stimulation induced tACS offline effects. These findings support the idea that tACS affects cortical excitability only during online application, at least when delivered on the scalp overlying M1, thereby contributing to the development of effective protocols that can be applied to clinical populations.

## Introduction

Transcranial alternating current stimulation (tACS) is a relatively new technique that can effectively modulate oscillatory brain activity through weak external alternating current at specific frequencies^1–3^. This effect is supported by research in animals^3,4^ and humans^1,2^, as well as by computational modelling^5^. In addition, a bulk of evidence has shown that tACS affects motor^6–9^, sensory^2,10,11^ and cognitive functions^12–15^. The possibility of enhancing cognitive functions promoted growing interest in tACS in the last decade^16^.

However, there is still a lack of understanding about the exact mechanisms that modulate cortical activity as a function of tACS administration^17,18^. These effects are in general mixed and controversial, since they depend on a number of stimulation parameters, such as stimulation duration, intensity, electrodes montage and size, stimulation phase and frequency^17,19,20^. Therefore, further investigation is needed to reach a comprehensive understanding of tACS effects. One of the main controversial issues is whether transcranial electrical stimulation (tES) (and tACS in particular) exerts its effects only when delivered online, that is, during ongoing stimulation, only offline (i.e. after-effects), after the stimulation has ceased, or both^17,21–23^.

This question can be addressed with different methods, including functional magnetic resonance imaging (fMRI), electro- and magneto-encephalography (EEG and MEG, respectively) and transcranial magnetic stimulation (TMS). A number of studies explored the temporal dynamics of tACS effects using EEG and MEG, especially focusing on the visual cortex and related activity in alpha-band^1,24–27^. However, tACS induces very large (compared to intrinsic brain electrical activity) artefacts on the EEG and MEG signal^28^. These artefacts confound the results of online tACS studies where effects of neuromodulation were recorded using EEG and MEG, even though new approaches to tACS artefacts rejection are promising^29^.

Another widely used approach to study online and offline physiological effects of tACS is measuring TMS-induced motor evoked potentials (MEPs). When TMS-induced MEPs are used to measure effects of tACS, tACS is applied over the primary motor cortex. Amplitude of TMS-induced MEPs is used as a measure of corticospinal excitability and is free from EEG/MEG technical constrains. Thus, amplitude of TMS-induced MEPs gives the possibility to measure both online^7^ and offline^30^ effects of tACS.

The majority of tACS studies using TMS-induced MEPs to measure effects of tACS neuromodulation demonstrated prominent frequency-specific online effects^6,7,31,32^. For instance, 20 Hz (beta-band) stimulation increased corticospinal excitability (measured as TMS-induced MEPs amplitude) at rest, while other frequencies (i.e. in delta-, theta- or gamma-band) did not modulate MEPs size^6,7,33^. In addition, online tACS effects are also state- and phase-dependent, meaning that different tACS frequencies induce different effects depending on the brain state^6^ and on the relative phase of the externally-delivered current^8,34,35^.

Reports on tACS offline effects are inconsistent and less frequently observed. For instance, in one of the first published tACS studies, Antal et al.^30^ applied tACS to the primary motor cortex (M1) at 1, 10, 15, 30 and 45 Hz and found no offline effects examined by TMS-induced MEPs. Later studies also revealed that the offline effects of tACS on MEPs size are short-lived^36^, or absent^35,37–40^. However, there is some evidence for considerable offline effects on the peak-to-peak MEPs amplitude after tACS administration^41^. Supposedly, weak tACS offline effects are observed as a function of task-related activity and frequency of stimulation. For instance, it was shown that when the stimulation is combined with a simultaneous active motor task, physiological and behavioral effects outlast the stimulation period^42^. Other evidence showed that tACS induced offline effects on MEPs size when delivered at *ripple* frequencies^43^ with 140 Hz^44–46^, 250 Hz^45^, 333 Hz^47^ or even higher frequencies such as 1, 2 and 5 kHz^48^. In summary, the dynamics of online and offline tACS effects are still a matter of debate. In order to address this issue, we conducted two single-blind experiments.

In Experiment 1, we tested online effects of tACS on the motor corticospinal output at rest by using a combined single pulse TMS-tACS approach^6,7,31^. Our dependent variable was the amplitude of TMS-induced MEPs recorded from the contralateral first dorsal interosseous muscle (FDI)^49^. In Experiment 2, we explored offline tACS effects by recording MEPs after the termination of the stimulation. In both experiments, tACS was delivered at 10 Hz (alpha-band) and 20 Hz (beta-band), and a sham (placebo) stimulation was included as control condition. The choice of alpha and beta frequencies was due to their physiological role in the sensorimotor system^50,51^. Previous evidence showed that online tACS of M1 delivered at the beta frequency modulates the corticospinal output, supposedly by means of resonance effects at rest^7,8^. Therefore, in the current study we expected an entrainment effect by tACS and consequently a corticospinal increase with 20 Hz stimulation compared to 10 Hz and sham (control conditions) and only for the online protocol (Experiment 1), i.e. we expected to find online but not offline tACS effects.

We ran two separate experiments to avoid confounding by possible carryover effects from combination of tACS and TMS, since the effect of combined tACS and TMS is not yet well explored. Moreover, we wanted two different groups of subjects to be assigned to each experiment in order to isolate further issues, such as number of experimental sessions that might be induce fatigue and lack of motivation, that could potentially interfere with the experimental output.

## Experiment 1

### Methods

#### Subjects

Participants were 24 right-handed (by self-report) volunteers (15 females, age range: 18–32, mean: 21.9, SD: 4.2). Participants had no personal or family history of neurological and psychiatric disorders and denied alcohol and drugs consumption in the days before the experiment. All participants gave informed written consent to partake in the study and received financial compensation after the experiment. We informed the participants that even if they would have decided to quit the experiment before the end, we would have paid them financial compensation. The study conformed to the ethical guidelines of the Declaration of Helsinki, and the experiment was approved by the Ethical Committee of the National Research University Higher School of Economics, Moscow. During the experiment participants sat comfortably in a reclining chair keeping their right arm relaxed.

#### TMS

Neuronavigated TMS was delivered over the left M1 via a MagPro X100 (MagVenture, Farum, Denmark) stimulator equipped with a C-B60 butterfly induction coil (75-mm wing outer diameter radius) to produce biphasic TMS pulses. The coil was held tangential to the scalp, with the handle pointing backwards and laterally, angled at 45° from the midline sagittal axis of the participant’s head. A frameless neuronavigation system (Localite TMS Navigator, Localite GmbH, Sankt Augustin, Germany) was used for MRI-guided navigation based on individual T1 weighted MR scans, allowing hot spot localization and stability of the coil position across the stimulation blocks during the whole experimental session.

The M1 hot spot was defined as the point on the scalp corresponding to the optimal coil position able to elicit reliable MEPs from the right FDI muscle based on the online EMG signal acquisition. Once the hot spot was found, the resting motor threshold (rMT) was measured using a staircase procedure until the minimal intensity of stimulation able to induce 50 µV peak-to peak amplitude of MEP in the 50% of cases (5 out of 10 MEPs) was found^49^. TMS intensity used during the experimental tasks was then fixed at 110% of the left (dominant) rMT.

Surface EMG activity was recorded from the right FDI muscle using disposable adhesive surface electrodes (EB Neuro S.p.A., Florence, Italy) placed in a belly-tendon montage with the BrainAmp (Brain Products GmbH, Munich, Germany) DC amplifier (sampling rate: 5 kHz).

#### tACS

tACS was delivered by a battery driven current stimulator (BrainStim, EMS Medical, Bologna, Italy) with two saline-soaked sponge electrodes (5 × 7 cm). The target electrode was placed over M1 (hot spot for FDI muscle, as determined by TMS) while the reference electrode was placed over the ipsilateral shoulder. The electrode over M1 was fixed with a rubber band. Impedances were kept below 10 kΩ. tACS (without DC offset) was delivered at 1000 μA (peak-to-peak) with a maximum current density of 14.3 μA/cm^2^ per electrode. Fade-in and fade-out times were set to 10 s. 10 Hz and 20 Hz tACS protocols were applied online for a total of 4 min per block (see “Procedure” below). Sham stimulation consisted of 30 s of low frequency transcranial random noise stimulation (tRNS) between 0.1 and 100 Hz (fade-in/fade-out: 10 s; intensity: 1000 μA; maximum current density 14.3 μA/cm^2^). We chose low-frequency tRNS to include the same frequencies used in the experimental tACS protocols (10 Hz, 20 Hz). Low frequency tRNS was used as reliable sham for tACS in previous studies^32,36,52^ and did not induce tangible offline effects on M1 excitability^53^. Finally, tRNS produces similar scalp sensations (e.g. itching, tingling) to those induced by tACS^54^. Participants were blind to the frequency of stimulation, as confirmed by a questionnaire administered after the experiment (see section “Questionnaire” for details).

#### Combined tACS-TMS

We adopted a combined tACS-TMS approach as in previous studies from our laboratory^6,7,31^. This procedure is well consolidated and involves finding an optimal hotspot for recording FDI muscle activity, placing the saline-soaked sponge target electrode of tACS above the hotspot, and adjusting the TMS coil position over the tACS electrode to measure the rMT according to the combined montage^31^. All blocks of MEPs (including baselines) were recorded after the placement of the sponge electrode. Moreover, to further adjust the position of the coil with the combined tACS-TMS approach, a new TMS hotspot was determined due to the possible altered magnetic field distribution after placing the tACS electrode^31^.

#### Procedure

**Figure 1:**
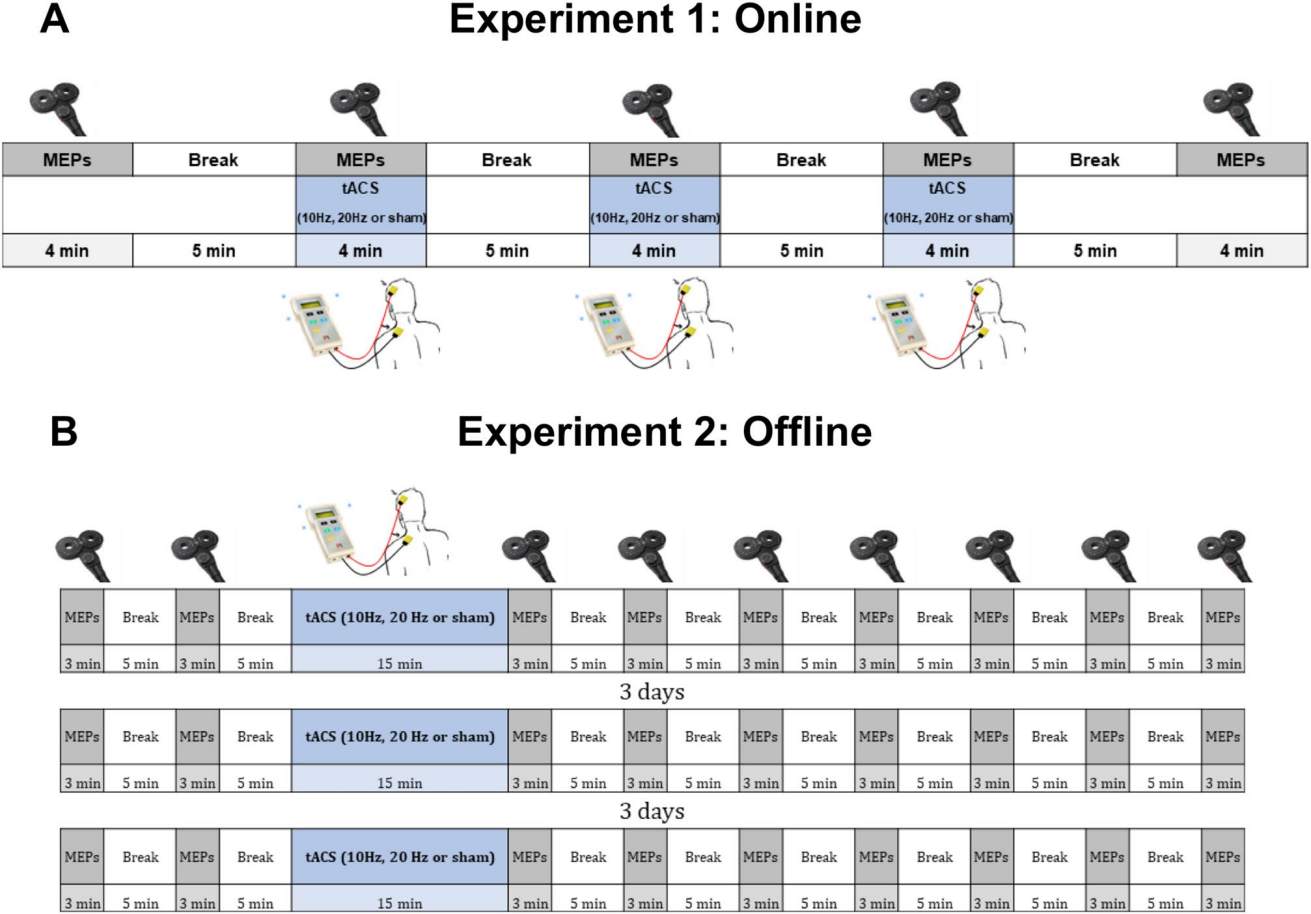
Experimental procedures. (**A**) Time course of tACS-TMS session for Experiment 1 (online). Two block of MEPs, PRE and POST, were recorded before and after three combined tACS-TMS blocks. tACS conditions (10 Hz, 20 Hz, sham) were accompanied by TMS-induced measurement (combined tACS-TMS). (**B**) Time course of tACS-TMS sessions for Experiment 2 (offline). Each session consisted of two blocks of MEPs before and seven blocks of MEPs after tACS. tACS conditions (10 Hz, 20 Hz, sham) were randomized across three sessions, that were separated by at least three days.

One block of baseline MEPs was recorded before tACS activation (PRE). We then recorded three blocks of MEPs during simultaneous tACS delivered at 10 Hz, 20 Hz or sham. Blocks of stimulation were randomized and counterbalanced. MEP blocks were divided by 5 min breaks (Fig. 1A). Average number of MEPs for each block was 23.9 (SD = 4.9). To ensure a reliable baseline and to control for any carryover effect of online stimulation, a further control block without tACS stimulation (POST) was recorded after tACS administration. Both PRE and POST blocks were recorded with the tACS headset on. The experimental session lasted for 40 min (Fig. 1A). The total amount of time required for the combined tACS-TMS setup and experimental session was around 2.5 h.

#### Questionnaire

At the end of the experiment volunteers filled out a questionnaire about side sensations felt during tACS application. We used a Russian version of the questionnaire by Fertonani, Ferrari and Miniussi^54^ which included items about itching, pain, burning, heat, pinching, metallic taste, fatigue and other sensations, in addition to their duration and localization. Moreover, participants were asked to guess whether active or sham stimulation was delivered in each of the three blocks.

#### Data analysis

Data analysis for signal processing was performed using MATLAB R2014a (The MathWorks Inc., Natick, MA, USA) while statistical computing was performed using R 3.6.0 (The R Project for Statistical Computing, Vienna, Austria). EMG activity was filtered with a high-pass filter and a notch filter (50 Hz) to remove power-line noise. MEP peaks were identified inside a 20–62 ms time window from the TMS stimulus, a range to detect all MEPs for each subject based on visual inspection^55^.

Artifact rejection was semi-automatic. First, artifact MEPs were manually rejected through visual inspection. This manual rejection procedure, which was blind to the experimental conditions, consisted of deleting MEPs with noise and muscle activity (including all activity with amplitude higher than > 0.1 mV) in the 300-ms preceding the TMS pulse, and with an inter-stimulus interval of less than 2 s. Then, MEPs were automatically discarded if they met at least one of these criteria: (1) peak-to-peak amplitude less than 50 µV, (2) latency jitter more than 2 ms from a median latency in the baseline condition, and (3) peak amplitude (negative or positive peak of MEP) less than the amplitude of the noise outside the 20–62 ms time window. The total percentage of discarded MEPs was 3.95%. Peak-to-peak amplitudes were transformed with a natural logarithm transformation and 10% trimmed mean logarithmic amplitudes for each block were calculated^6,34,56,57^. This procedure was used to normalize the distribution of amplitude data and reduce the heteroscedasticity, therefore allowing inter-individual comparisons.

In order to test any change after tACS administration we compared PRE and POST conditions using two-tailed t-tests. Since comparison revealed no differences (see “Results” section), we defined a baseline as an average of the PRE and POST blocks. Then logarithmized and averaged data for each subject’s condition were normalized as a change in percentage compared to the baseline. Then, we conducted a one-way repeated measures ANOVA with stimulation condition as the independent variable (20 Hz, 10 Hz, and sham), and the logarithmized MEPs change as the dependent variable. We applied the Greenhouse–Geisser correction to compensate for sphericity violations, and performed post hoc Tukey HSD comparisons in the presence of significant interactions.

Subjective scores (individual ratings for itching, pain, burning, heat, pinching, metallic taste, fatigue, other sensations) were collapsed into a unique questionnaire score by summation of all sensation ranks. The subjects’ scores were compared between stimulation conditions (10 Hz, 20 Hz, sham) using the Friedman test. In addition, subjective scores were correlated with logarithmized MEPs change for all conditions using the Pearson correlation coefficient. All p-values were corrected for multiple comparisons using Bonferroni correction, if appropriate.

### Results

#### Baseline comparisons

Differences between PRE and POST baselines were not significant: *t*(23) = 0.20, *p* = 0.840, *d*_*z*_ = 0.04.

#### tACS frequency effects

**Figure 2:**
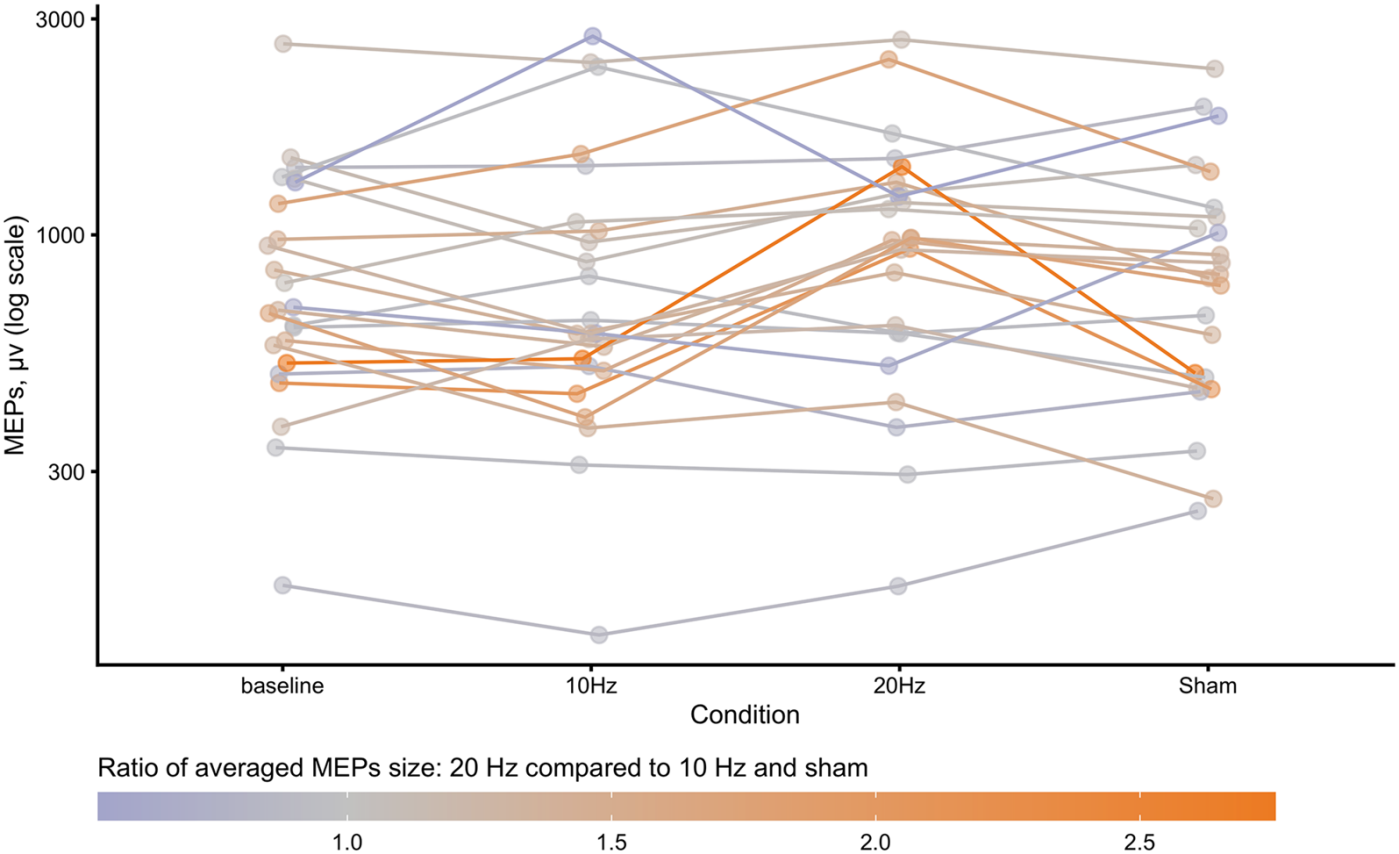
Individual logarithmized MEPs (in µV) measured during the baseline (averaged blocks PRE and POST) and during three tACS conditions (10 Hz, 20 Hz, sham) in Experiment 1 (online). Colors represent the ratio between individual MEPs size during 20 Hz compared to the average of individual MEPs size during 10 Hz and sham.

**Figure 3:**
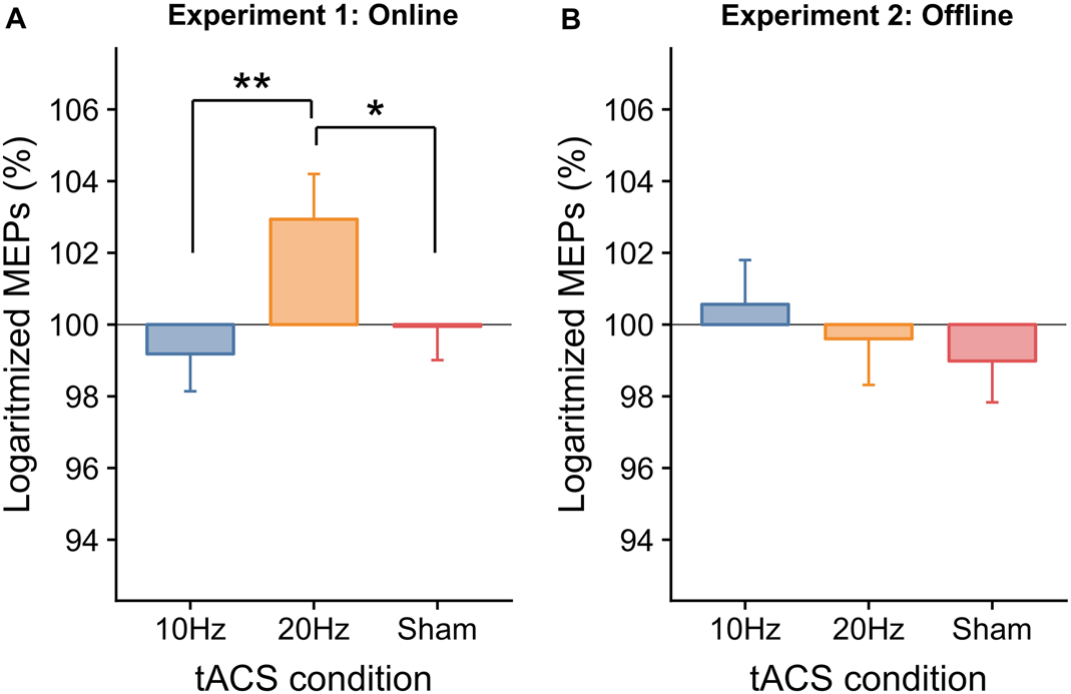
Frequency-dependent tACS effects on the primary motor cortex excitability for Experiment 1 (online) and 2 (offline). (**A**) Relative change in % of logarithmized MEPs compared to the baseline in Experiment 1 for three tACS conditions (10 Hz, 20 Hz, sham). (**B**) Averaged across time relative change in % of logarithmized MEPs compared to the baseline in Experiment 2 (offline) for three tACS conditions (10 Hz, 20 Hz, sham). Error bars indicate sem. *p < 0.05; **p < 0.01.

The one-way ANOVA yielded significant differences across the three stimulation conditions, *F*(2, 46) = 4.35, *p* = 0.019,  ${\eta }_{p}^{2}$ =  0.16 (see Fig. 3A). Post-hoc comparisons showed that MEPs change during 20 Hz tACS (average raw MEPs size: 1043 µV ± 613 µV) was higher compared to 10 Hz (p = 0.005, average raw MEPs size: 916 µV ± 696 µV) and sham (p = 0.026, average raw MEPs size: 908 µV ± 546), which did not differ from one another (p = 0.570). This result supports the notion that tACS induces robust frequency-dependent effects when delivered online. See Fig. 2 which represents individual MEPs size for different stimulation conditions.

#### Questionnaire

The questionnaire administered at the end of the experiment revealed that 18 participants judged the 20 Hz as the active condition (75%), 16 participants marked that 10 Hz was active (67%) and 15 marked sham as the active condition (62%): $${\chi }^{2}$$(2, n = 72) = 0.89, *p* = 0.639. The Friedman test showed no significant differences across conditions in tACS-induced sensations ($${\chi }^{2}$$(2) = 1.38, *p* = 0.502), confirming successful blinding. We found no significant correlation between MEPs size and tACS-induced sensations (10 Hz, $$r$$ = 0.13, *p* > 0.999; 20 Hz, $$r$$ = − 0.26, *p* = 0.650; sham, $$ r$$   =  − 0.13, *p* > 0.999, see Supplementary Figure 1A).

## Experiment 2

### Methods

In Experiment 2, we recruited 19 new right-handed (by self-report) participants (10 females, age range: 18–27, mean: 21.1, SD: 2.7). Gender distribution for two experiments did not differ significantly (${\chi }^{2}$(1) = 0.12, *p* = 0.734) as well as age distribution (*t*(39.28) = − 0.78, *p* = 0.439). Participants had no personal or family history of neurological and psychiatric disorders and denied alcohol and drugs consumption in the days before the experiment. All participants gave informed written consent to partake in the study and received financial compensation after the experiment. We informed the participants that even if they would have decided to quit the experiment before the end, we would have paid them financial compensation. The study conformed to the ethical guidelines of the Declaration of Helsinki, and the experiment was approved by the Ethical Committee of the National Research University Higher School of Economics, Moscow. During the experiment participants sat comfortably in a reclining chair keeping their right arm relaxed.

One male participant was excluded from analysis due to technical issues. According to an a priori power analysis using G*Power 3.1.9.4^58^ sample size was determined to be not less than 15 (using estimate of ${\eta }_{p}^{2}$ = 0.16 and correlation among variables 0.25 from the results of Experiment 1, for alpha = 0.05 and 0.8 target power). We recruited more subjects than needed to account for possible drop-outs since the experiment is across multiple days. As revealed by post hoc power analysis, achieved statistical power that were included in the analysis was therefore higher and equal to 0.9.

**Figure 4:**
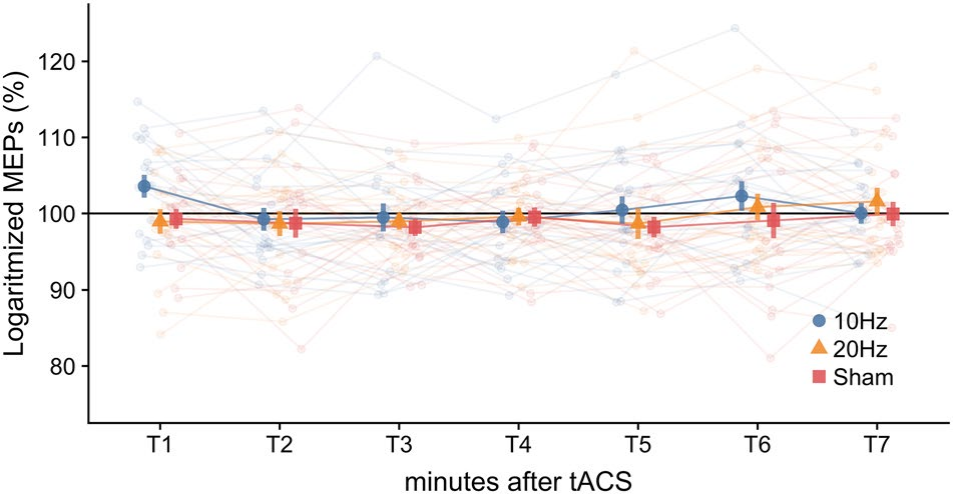
Time course of the primary motor excitability changes in % to the baseline for Experiment 2 (offline) for three tACS conditions (10 Hz, 20 Hz, sham) measured as logarithmized MEPs. Transparent lines represent individual dynamics of the primary motor excitability changes for each condition. Error bars indicate sem.

Methods of Experiment 2 were identical to the Methods of Experiment 1, with some adjustments to allow the measurement of tACS offline effects: (1) MEPs were recorded after the termination of the stimulation, which lasted 15 min. In particular, seven blocks of MEPs were recorded from the FDI muscle, divided by a 5 min break. Two blocks of MEPs (with 5 min break in between) were recorded before tACS to obtain a reliable baseline. Average number of MEPs per block: 21.0; SD: 3.3; 10.8% of MEPs were discarded due to artefact rejection procedure that was identical to procedure in Experiment 1, (2) Each stimulation frequency was tested on a different day as in typical offline tES/TMS protocols. Stimulation protocols were randomized and counterbalanced across the days. Therefore, the experiment consisted of three sessions, divided by 3 days, with duration of 82 min each. To verify that there were no statistically significant differences between baseline conditions, we conducted a one-way repeated measures ANOVA with factor Stimulation Condition (20 Hz, 10 Hz, and sham) and a one-way way repeated measures ANOVA with factor Order (the first, the second, and the third experimental session) on logarithmized MEPs. The total amount of time required for the experimental setup and experimental session was around 3 h, (3) To estimate the time-course of tACS offline effects, we analyzed the effects of each stimulation frequency as a function of the number of blocks elapsed after tACS administration (see Fig. 1B), (4) The questionnaire was delivered in the end of each session. In the end of the experiment (after the third session), participants were asked to guess whether active or sham stimulation was delivered in each of the sessions.

### Results

#### Baseline differences

One-way repeated measures ANOVA on baselines with factor Stimulation session (20 Hz, 10 Hz, and sham) on logarithmized MEPs did not show statistically significant differences across sessions (*F*(2, 34) = 0.01, *p* = 0.988, ${\eta }_{p}^{2}$ < 0.01). However, one-way repeated measures ANOVA with factor Order (the first, the second, and the third session) for the baselines, showed statistically significant differences between sessions (*F*(2, 34) = 3.45, *p* = 0.043, ${\eta }_{p}^{2}$ = 0.17). There was a significant increase in averaged logarithmized MEPs size from the first to the third session (p = 0.009) regardless of the tACS condition. All other differences were not significant (p > 0.05).

#### tACS frequency effects

We run a two-way ANOVA with factors Stimulation Condition (three levels, 20 Hz, 10 Hz and sham) and Block (seven levels, corresponding to seven blocks after the stimulation elapsed) on the logarithmized MEPs change. The main effects of Stimulation Condition (*F*(2, 34) = 0.47, *p* = 0.630, ${\eta }_{p}^{2}$ = 0.03) and Block (*F*(6, 102) = 1.53, *p* = 0.175, ${\eta }_{p}^{2}$ = 0.08), and their interaction (*F*(12, 204) = 0.98, *p* = 0.467, ${\eta }_{p}^{2}$  =  0.05) were not significant (see Figs. 3B and 4). This result supports the hypothesis that tACS does not induce frequency-specific offline effects.

#### Questionnaire

The questionnaire administered at the end of the experiment revealed that 9 participants believed that the sham condition was the active one (47%), while 12 participants marked 10 Hz (63%) and 20 Hz (63%) conditions as active conditions, ${\chi }^{2}$(2, n = 57) = 1.30, *p* = 0.523. No significant differences emerged when comparing subjective feelings as a function of stimulation condition (${\chi }^{2}$(2) = 2.39, *p* = 0.302), confirming a successful blinding procedure. For Experiment 2, MEPs changes were averaged across blocks and correlated with the unique questionnaire score (as a sum of individual rating) using the Pearson correlation coefficient. We found no significant correlations for 10 Hz   (*r* = 0.48, *p* = 0.209), 20 Hz  (*r* = − 0.59, *p* = 0.062) and sham $(r$ = 0.00, *p* > 0.999) (see Supplementary Fig. 1B).

## Discussion

The current study aimed to examine online and offline effects of tACS at 10 and 20 Hz on the excitability of M1 in healthy young adults. We tested the hypotheses that tACS modulated the activity of the primary motor cortex (M1) in a frequency-dependent manner^59^ and that such effects were limited to the time when tACS is delivered (i.e. online tACS effects vs offline tACS effects). In Experiment 1, we replicated previous findings by showing a small but reliable 20 Hz tACS online effects on the amplitude of TMS-induced MEPs^6,8,33,36,57^, compared to 10 Hz tACS and sham. In Experiment 2, we did not find any significant MEPs change after tACS at any frequency and interval after the termination of the stimulation, suggesting little-to-no offline effects of tACS on motor excitability.

Our findings contribute to explanation of some inconsistencies in the literature regarding tACS-induced effects on M1 excitability^6,8,34,57,60^. One possible explanation for mechanisms of tACS effects on neural processing involves the notion of entrainment. Entrainment is the temporal alignment of endogenous rhythmical brain activity to exogeneous alternating current^61^. According to this notion, if modulatory effects of tACS are mediated by entrainment, then these effects would last only while exogenous stimulation is present and wane quickly after tACS cessation. Previous studies so far indirectly supported this idea. For example, several studies found prominent 20 Hz stimulation effects on M1 excitability measured as TMS-induced MEPs during the administration of tACS^6–8^, although others did not find tACS-induced MEPs amplitude changes after 20 Hz tACS administration^37,38^. Another source of support to the idea of the entrainment hypothesis comes from recent studies of gamma-tACS neuromodulation of the primary motor cortex^40^. Even though there is no evidence that tACS in the gamma band modulates single pulse MEPs size either online or offline^7,30,39,62,63^, it modulates short-interval cortical inhibition (SICI) only during but not after tACS application^39,40,63^. This effect can be explained by the entrainment of GABA-A-ergic interneurons inside the primary motor cortex. In our study, we aimed at comparing offline and online effects of beta-tACS and revealed that tACS during resting state is effective only when delivered online, thus supporting the entrainment hypothesis.

However, there are several studies that show offline tACS effects on the M1 excitability. For instance, offline effects were found using 140 Hz^44–46^, 250 Hz^45^ and even higher frequency stimulation: 1 kHz, 2 kHz and 5 kHz^48^. High-frequency tACS may have different underlying mechanisms^18^, for example, the stochastic resonance effect as hypothesized for tRNS stimulation^64^. This idea is indirectly supported by the results of a recent study that showed a correlation between the effects of tRNS and high-frequency tACS^44^. In addition, tRNS induced offline effects on the M1 excitability only at high-frequency (101–640 Hz), but not at low-frequency (0.1–100 Hz) ranges^53^. Therefore, the underlying mechanisms of low-frequency and high-frequency tACS and tRNS may differ in terms of modulation of the ongoing brain activity. Additionally, scarce evidence showed that offline tACS effects can be induced when stimulation is accompanied by loading subjects with an active motor task^42^, in such a way that tACS could affect motor learning by means of entrainment during the stimulation. Active motor learning during tACS could influence brain excitability even after stimulation cessation. Such evidence is limited because the majority of tACS studies of the M1 excitability applied tACS during resting state. Thus, the entrainment hypothesis provides a parsimonious explanation of our results as well as results of previous studies on tACS M1 neuromodulation.

It should be mentioned that a few studies showed no online effects of beta-tACS on the primary motor cortex excitability measured with TMS-induced MEPs^8,35,39,62,65,66^. One explanation for the lack of statistically-significant increases of MEPs size during beta-tACS is the relatively small effect size combined with a small sample size, leading to low statistical power and high number of type II statistical errors. Another explanation takes into account differences in stimulation protocols, both for tACS and TMS. For example, some studies used low tACS intensity or adjusted intensity to avoid potential neurosensory side effects: 0.69 mA (average)^65^, 0.71 mA (average)^35^, 0.78 mA (average)^39^. These intensities are lower than those used in other studies where online beta-tACS effects were found^6,7,32,42^. Alternatively, the lack of effects in the studies mentioned above can be indeed explained by the application of monophasic TMS instead of biphasic TMS as used in our study. It can be hypothesized that biphasic TMS-induced MEPs is a more sensitive method to measure beta-tACS neuromodulation. Biphasic TMS may activate an extra pool of neurons in addition to those stimulated by monophasic TMS^67^, especially in combination with tACS. More systematic studies of beta-tACS effects on MEPs obtained with monophasic and biphasic TMS will shed light on mechanisms of tACS neuromodulation. Of note, one study with biphasic TMS failed to observe differences of beta-tACS online effects compared to alpha-tACS and sham^66^. These results are somewhat surprising because of the higher intensity of tACS (1.5 mA) combined with relatively small (3 × 3 cm) electrodes. One possible explanation for the lack of effect is the non-monotonic dose–response function of tACS intensity on the effect of neuromodulation, i.e. higher intensity stimulation may have lower or even opposite neuromodulation effect. Some evidence for the non-monotonic dose–response function of stimulation intensity comes from transcranial direct current stimulation (tDCS) studies^68,69^ and ripple frequency tACS^46^. Further research is needed to elucidate the controversial effects of online tACS likely due to manipulation of stimulation parameters, sample size and task/protocol.

There is an implicit assumption that different tES methods have similar mechanisms, thus having similar effects on brain activity, including the time-course of these effects. In the modern history of tES application, tDCS was the first method to appear. More recently, tACS and tRNS were developed as variations to tDCS and similar parameters of stimulation were used for their application. Indeed, even using the umbrella term “tES” for three electrical stimulation methods with potentially different mechanisms of action might be misleading.

However, the neuromodulatory mechanisms of tDCS, tRNS and tACS are different. In particular, at a cellular level, the application of tDCS induces neuronal depolarization during anodal stimulation and neuronal hyperpolarization during cathodal stimulation^70^. The injection of weak direct electrical current produces a change of the resting membrane potential that consequently can increase or decrease spontaneous firing rates accordingly to the stimulation polarity^71^. These effects are commonly reported offline both in the motor and cognitive domains^23,72,73^. Indeed, with a tDCS-TMS combined approach (similar to the tACS-TMS combined approach applied in the current study), it has been demonstrated that offline tDCS modulated M1 excitability of subjects at rest^74^. On the other hand, the time course of tDCS effects may be task-dependent or at least may vary depending on the time of the stimulation administration. As for tACS, the phase of the electrical current alternates between positive and negative voltages. Therefore, unlike tDCS, tACS induces a physiological entrainment through specific frequencies of stimulation^18^. Of note, it has been recently shown that the effects of tACS over the M1 may be transcutaneous meaning that the effects of the stimulation may be driven by cranial nerves rather than by specific activation of M1 cortical neurons^75^. This evidence is isolated, but it raises an important point regarding the neural mechanisms that subtend the effects of tACS and potentially of other tES techniques.

Thus, distinct tES methods have distinct mechanisms of neuromodulation. Results obtained for one tES method may not apply to other methods. Different authors emphasize the importance of systematic investigation of stimulation parameters on neuromodulation as in the current study^17,21,76,77^.

The current study provides evidence that tACS effects on the M1 excitability are elicited during stimulation and not offline thus supporting the entrainment hypothesis. Some limitations should be considered. There are few differences between the two experiments: time of stimulation delivering, sample size and number of sessions. However, firstly, differences in duration of tACS could not have affect neuromodulation effectiveness unless tACS duration has a non-linear effect on the M1 excitability. Secondly, statistical power analysis demonstrates that sample size for the Experiment 2 was sufficient. Finally, results of the Experiment 2 provide evidence that carryover effects of tACS, if any, are negligible. On the other hand, frequency, intensity of stimulation, electrodes montage, tACS-TMS protocol (including stimulation and recording devices) and all other parameters were the same for both experiments. Thus, different results in Experiment 1 and 2 are most likely due to the fact that we used online tACS protocol for Experiment 1 and offline tACS protocol for Experiment 2. Therefore, we assume that our study provides valid contribution to the field.

The use of tACS is growing fast in brain research. The idea to potentially entrain endogenous oscillatory activity by means of external sinusoidal potentials is very appealing. However, efficient application of tACS is premature without in-depth understanding of the mechanisms underlying tACS neuromodulation.

We believe that disentangling the temporal dynamics of tACS effects is crucial for the clinical application of this technique. Our findings pave the way for the development of tailored stimulation protocols and shed a light on the mechanisms of tACS neuromodulation.

## Supplementary Information

Supplementary Information.

## Data Availability

The dataset analyzed during the current study is available from the corresponding author on reasonable request.
