# Supplementary material for: Online and offline effects of transcranial alternating current stimulation of the primary motor cortex
Source: Sci Rep. 2021 Feb 16;11:3854. doi: 10.1038/s41598-021-83449-w (PMC7887242; doi:10.1038/s41598-021-83449-w)

Online and offline effects of transcranial alternating current stimulation of the primary motor cortex

Ivan Pozdniakov[1], Alicia Nunez Vorobiova[1], Giulia Galli[2], Simone Rossi[3], Matteo Feurra*[1,4]

[1]Centre for Cognition and Decision Making, Institute for Cognitive Neuroscience, National Research University, Higher School of Economics, 101000, Moscow, Russia.

[2]Department of Psychology, Kingston University, Penrhyn Road, Kingston Upon Thames, KT1 2EE, United Kingdom

[3]Department of Medicine, Surgery and Neuroscience, Siena Brain Investigation & Neuromodulation Lab (Si-BIN Lab.), Unit of Neurology and Clinical Neurophysiology and Section of Human Physiology, University of Siena, Italy, 53100

[4]National Research University, Higher School of Economics, 101000 Moscow, Russia

Corresponding author: Matteo Feurra (mfeurra@hse.ru; matfeu@gmail.com), Centre for Cognition and Decision Making, Institute for Cognitive Neuroscience, National Research University, Higher School of Economics, 101000, Moscow, Armyanskiy per. 4, c2 - Room 404. Tel. +79104688329.

SUPPLEMENTARY INFORMATION

Supplementary Figure 1 . Scatter diagram of the tACS-induced sensations (Discomfort) vs. individual logarithmized MEPs changes to the baseline (in %). (A) Experiment 1 (online): sum of individual rating for tACS-induced sensations vs. individual logarithmized MEPs changes to the baseline during combined tACS-TMS. (B) Experiment 2 (offline): sum of individual rating for tACS-induced sensations vs. individual logarithmized MEPs changes to the baseline were averaged across time.

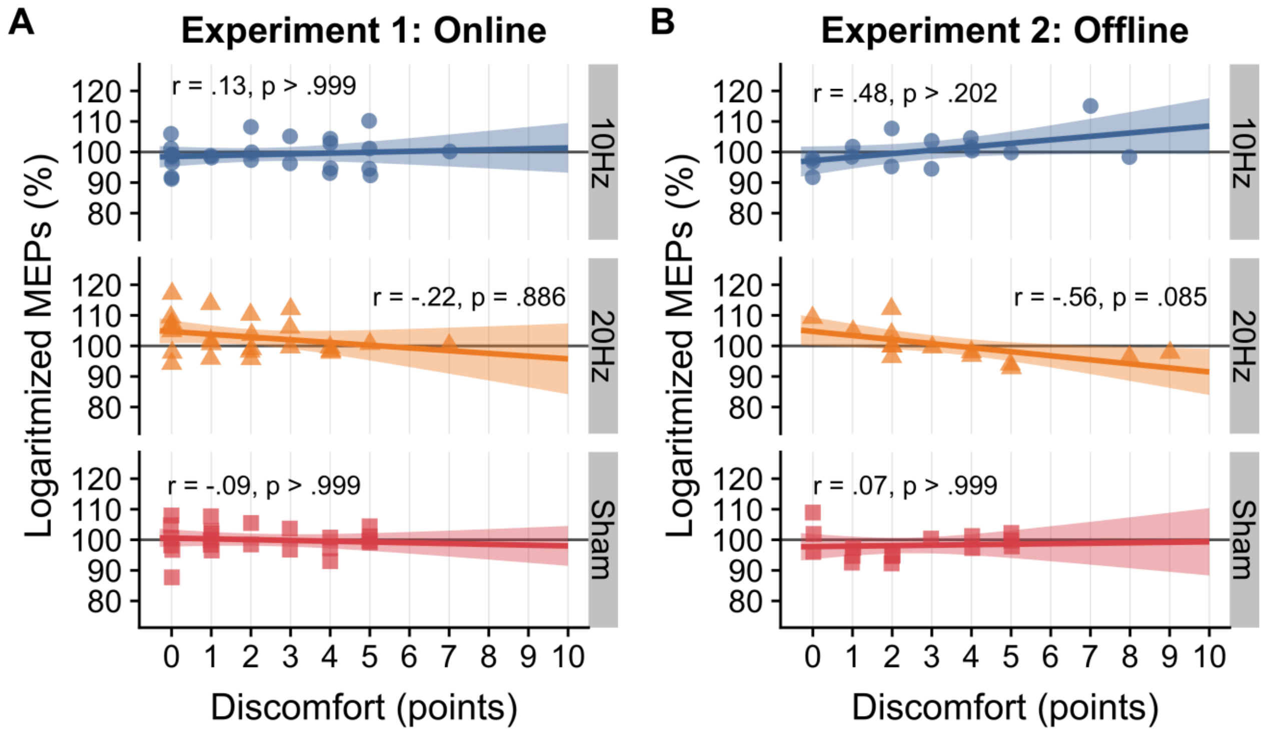

Supplement: Supplementary file 1 — Supplementary Information. [file 41598_2021_83449_MOESM1_ESM.pdf]
